# Malaria Epidemiology in Kilifi, Kenya during the 21st Century: What Next?

**DOI:** 10.1371/journal.pmed.1002048

**Published:** 2016-06-28

**Authors:** Lorenz von Seidlein, Jakob Knudsen

**Affiliations:** 1 Mahidol-Oxford Tropical Medicine Research Unit (MORU), Mahidol, Thailand; 2 The Royal Danish Academy of Fine Arts, Schools of Architecture, Design and Conservation, Copenhagen, Denmark

## Abstract

Lorenz von Seidlein and Jakob Knudsen discuss the changes in malaria incidence recorded at a single site in Africa over 25 years, along with future implications for disease prevention.

In this issue of *PLOS Medicine*, Polycarp K. Mogeni and colleagues present a unique dataset collected from 69,104 children aged from 3 months to 13 years admitted over the last 25 years to Kilifi County Hospital, Kenya. In 1998, 56% of the children were admitted with malaria, presumably mostly caused by *Plasmodium falciparum*. By 2009, just 7% of the children were admitted with malaria. After the devastating impact of a very high malaria burden at the end of the last century, malaria-related morbidity and mortality declined dramatically in Kilifi, as it had done in many, but not all, parts of sub-Saharan Africa during the first decade of the 21st century [1]. Insecticide-treated bednets (ITNs) have played a central role in the control of malaria [2], although many other concurrent events—including rapid economic development of the continent and the introduction of artemisinin-containing combinations as first-line treatment for uncomplicated malaria—must have played important contributing roles.

In Kilifi, the downward trend in hospital admission with malaria reached its nadir in 2009 and then slowly started to increase again. This observation, which is not limited to Kilifi [3], has caused concern. Are established control measures no longer working? Is resistance against insecticides and antimalarials cancelling earlier gains, with malaria morbidity and mortality returning to previous levels? Mogeni and colleagues provide a convincing, alternative explanation. During the rapid decrease in malaria incidence up to 2009, lower rates of childhood exposure to *P*. *falciparum* infections meant that fewer children could acquire immunity to this pathogen. Over the following years, these immunologically naïve children in a setting of much reduced but still substantial risk of exposure eventually became infected. This epidemiological shift is shown by the increase in the mean age of children admitted with *P*. *falciparum* infections, which increased gradually from 20.2 months in 1990 to 45.3 months in 2014. With a further decrease in malaria incidence, the mean age of malaria patients seems likely to continue to increase.

Although malaria burden is much reduced, it remains unacceptably high; what can be done to reduce malaria transmission further?

Mogeni and colleagues illustrate nicely that the highest incidence of malaria is now in kindergarten and school-age children and no longer in infants and very young children. Malaria control programmes originating from the last century target infants, with a focus on adding malaria-related interventions to the expanded program on immunisation (EPI). Future malaria control initiatives will have to target older children. Seasonal malaria chemoprevention (SMC) is a welcome initiative in this direction [4]. Recommended across the Sahel subregion, SMC entails a complete treatment course of amodiaquine plus sulfadoxine/pyrimethamine (AQ+SP) given to children aged between 3 and 59 months at monthly intervals, beginning at the start of the transmission season, to a maximum of four doses during the malaria transmission season. But the malaria season in Kilifi is much less pronounced than in West Africa, and children would have to take monthly prophylaxis during most months to be protected all year round.

Mogeni and colleagues show that in Kilifi County, ITNs were highly protective; children who live in communities with high bednet use were less likely to present with *P*. *falciparum* infections, compared to children in communities with low bednet usage. Following the notion that bednets are a good thing and, therefore, more bednets must be even better, the authors propose universal coverage. The benefits of bednets have been repeatedly highlighted. Lengeler reviewed 18 randomised trials of ITNs and found that ITNs reduce the incidence of uncomplicated malaria by 48% compared to no net, and by 34% compared to non-impregnated nets. ITNs afford a 17% protection against mortality in children under 5 years [5]. Policymakers and donors have accepted the benefit of ITNs: between 2005 and 2015, more than 1 billion bednets were distributed in sub-Saharan Africa alone [6]. With the entire African continent’s population currently estimated at around 1.1 billion, on average, every person living on the African continent should have received 0.9 bednets over the last 10 years [7]. Even allowing for uneven distribution, does every person in a household need an individual net? In many malaria-endemic regions, a typical home has one or two small rooms shared by around five people. Two nets (e.g., one for adults and one for children) may provide adequate protection in such a setting. Assuming an average of five people per household, the average African household should have received 4.7 bednets over the last 10 years.

In Kilifi, 83% of respondents stated that they already use bednets; can the distribution of ever more bednets be a solution for the malaria problem in Kilifi? Having reached high levels of bednet ownership, the problem is now likely to be the use of bednets, not their availability. The current approach to assessing bednet use is asking respondents in home surveys whether they used bednets during the night before. There is an assumed correlation between the self-reported “have you/your child slept under a bednet last night” and the actual use of the bednet during each night of the malaria season, but there are no reliable data to confirm such an assumption. More likely, the self-reported responses are biased by participants’ inclination to provide what they believe is the desirable response. A more reliable answer could be gained from in-depth interviews, but such time-consuming methods are not suitable for large surveys.

There are a number of reasons why people do not use their bednets. Pulford and colleagues found that the negative effect on indoor climate was the most frequently stated reason for not using available bednets [8]. The indoor climate in the typical African home in the hot–humid zone is uncomfortable. The only relief comes from the occasional breeze; hence, ventilation is crucial. Bednets reduce airflow by more than 60% [9]. In such an environment, an uncomfortable room will become unbearable by hanging up a bednet. The residents have to choose between the discomfort caused by bednets or mosquito bites. Under these circumstances, the continued distribution of additional bednets is unlikely to increase bednet usage as hoped. Moreover, there is now reliable documentation for the abuse of ITNs for fishing [10]. In villages along Lake Tanganyika, Tanzania, 87% of households surveyed had used a mosquito bednet for fishing at some point [11].

What measures can be taken to increase the use of bednets for the intended purpose? Our review of factors that improve indoor climate in hot–humid zones found that the single most important factor is proper cross-ventilation. Elevating houses on stilts, using a bamboo or wooden floor, as is standard construction practice in Southeast Asia, and the opening of eaves and windows had the largest positive impact on indoor climate [12]. Surprisingly, elevated houses are difficult to find in rural sub-Saharan Africa, and substantial, open windows are the exception. Ideally, new housing should take these ideas into account, but the replacement of sub-optimal housing will take several generations. Meanwhile, remedial steps, such as the addition of open windows, ideally with mosquito screens, will be beneficial. To make an impact on malaria transmission in homes where no adequate windows are present, we propose that two windows should be installed for every new bednet distributed.

More effort should be made on community engagement and education to discuss novel architecture of new houses being built in Africa, to gradually integrate housing characteristics that improve ventilation while minimising vector entry. Elevating the house off the ground reduces mosquito bites and improves airflow [13]. Adding windows and using lighter or porous wall and floor materials such as bamboo will improve cross-ventilation and indoor comfort. Such improvements increase the likelihood that bednets are used throughout the night, every night. Housing improvements have contributed to the elimination of malaria from the United States and the control of malaria in Europe and are protective in sub-Saharan Africa [14–16]. Where outdoor biting anophelines play a major role in disease transmission, bednets are of less use. Before electricity and air conditioning became universally available in the southern states of the US, the screened porch played a central role in many homes. Providing the benefit of the cool evening breeze without the nuisance of mosquito bites, screened porches became a well-recognised architectural feature [17]. Perhaps related to the lack of cheap but robust screening materials, there has been little experience with screened outdoor spaces in malaria-endemic, tropical countries in rural sub-Saharan Africa.

Housing improvements will benefit all age groups and provide protection against malaria for adults as well as children [18,19]. Much progress has been made in Kilifi and other parts of malaria-endemic regions; novel approaches, such as the integration of housing improvements, are now needed to drive malaria to extinction.

## Abbreviations

AQ+SP: amodiaquine plus sulfadoxine/pyrimethamine
EPI: expanded program on immunisation
ITN: insecticide-treated bednet
SMC: seasonal malaria chemoprevention
